# Effect of patellofemoral joint overstuffing following total knee arthroplasty without patella resurfacing on clinical efficacy and related factors analysis

**DOI:** 10.1186/s13018-024-04899-2

**Published:** 2024-07-31

**Authors:** Feida Wang, Guohao Zhang, Xiaochun Wei

**Affiliations:** Department of Orthopedics, Second Hospital of Shanxi Medical University, Taiyuan City, 030001 Shanxi Province China

**Keywords:** Without patella resurfacing, Total knee arthroplasty, Patellofemoral joint, Overstuffing phenomenon, Influencing factors

## Abstract

**Objective:**

To analyze the influencing factors for patellofemoral joint (PFJ) overstuffing following total knee arthroplasty (TKA) without patella resurfacing, and explore the effect of PFJ overstuffing on clinical efficacy.

**Methods:**

A retrospective analysis was conducted on 168 patients with end-stage knee osteoarthritis who underwent TKA without patella resurfacing at our hospital between Match 2019 and September 2021. The clinical data of these patients were retrospectively analyzed. In this study, PFJ overstuffing was defined as a postoperative PFJ distance greater than 1 mm compared to the preoperative measurement. The occurrence of postoperative PFJ overstuffing was counted. The patients were divided into the overstuffing group (*n* = 109) and the non-overstuffing group (*n* = 59) to count the patellar thickness and thickness of femoral anterior condyle in all patients before and after surgery, and analyze the influencing factors for postoperative PFJ overstuffing in such patients. Patients were followed up for 2 years to compare the recovery time of postoperative pain, score of visual analogue scale (VAS) and flexion activity between the two groups.

**Results:**

There was no significant difference in patellar thickness between preoperative and postoperative measurements of the patients (*P* > 0.05). However, the thickness of the femoral anterior condyle and the PFJ distance after surgery increased significantly compared with those before surgery (*P* < 0.05). Among the 168 patients, 109 cases (64.88%) experienced PFJ overstuffing. The risk of PFJ overstuffing was higher in female patients than in male (*P* < 0.05). The preoperative thickness of the femoral anterior condyle in the overstuffing group was significantly smaller compared to the non-overstuffing group (*P* < 0.001). Compared with the non-overstuffing group, the overstuffing group had longer recovery time of postoperative pain (*P* < 0.05), and had lower flexion activity at 2 years after surgery (*P* < 0.001). However, no significant difference was found in VAS score between the overstuffing group and the non-overstuffing group at 2 years after surgery (*P* > 0.05). Spearman rank correlation analysis indicated females tend to have a lower preoperative thickness of the femoral anterior condyle (*r*=-0.424, *P* < 0.001), as well as a positive postoperative PFJ overstuffing (*r* = 0.237, *P* < 0.05). Furthermore, there was a negative correlation between preoperative thickness of the femoral anterior condyle and postoperative PFJ overstuffing (*r*=-0.540, *P* < 0.001).

**Conclusion:**

Following TKA without patella resurfacing, there is a high risk of PFJ overstuffing, particularly among female patients and those with a small thickness of the femoral anterior condyle. Therefore, special attention should be given to these high-risk groups during clinical treatment.

## Introduction

Total knee arthroplasty (TKA) is a highly successful surgical procedure aimed at alleviating pain and restoring function in patients suffering from end-stage knee osteoarthritis [1]. Despite its positive outcomes, the occurrence of complications such as patellofemoral joint (PFJ) overstuffing after TKA can lead to suboptimal clinical results, including severe anterior knee pain [2]. Patella resurfacing is a commonly employed technique to address the anterior knee pain caused by PFJ overstuffing; however, its necessity remains a subject of debate in the field of orthopedic surgery.

Advancements in biomechanics, prosthesis design, surgical instruments and technology have contributed to the remarkable success of TKA. Patellar replacement has become a routine operation in modern TKA, which can reduce the occurrence of postoperative reoperation and noise, and improve the function of knee joints [3]. However, the use of patellar replacement introduces the potential for serious complications, such as patellar maltracking, fracture, avascular necrosis, clunk and anterior knee pain [4], leading to a selective approach in its implementation. TKA is a highly precise procedure, and any deviations may have an adverse impact on the mechanical function of the knee joint, ultimately affecting patient prognosis. Unfortunately, research on overstuffing after TKA without patella resurfacing remains limited due to the historical focus on the patella and insufficient consideration of its relationship with the femoral side.

The concept of overstuffing primarily originated in the context of patellar replacement, referring to an increase in femoral anterior or posterior condylar offset compared to preoperative measurements, while PFJ overstuffing refers to the increased distance between anterior cortex of patella and femoral trochlear than before surgery [5] due to the use of non-individualized prosthesis or surgical technique. However, there is no golden standard of measurement technique to quantify this point [6, 7]. Overstuffing can result in various adverse consequences, including limited range of motion and anterior knee pain [8]. Overstuffing is not exclusive to patellar replacement but is also prevalent in non-patellar replacement procedures. The appropriate prosthesis size and precise alignment of the lower limb alignment have been identified as crucial factors influencing surgical outcomes. The anatomical structure of the distal femur and the non-individualized design of prostheses are significant contributors to overstuffing, which in turn leads to increased pain and limited knee joint flexion [9]. Consequently, the analysis of influencing factors for PFJ overstuffing is imperative in optimizing surgical planning and improving clinical efficacy.

However, only a small number of studies have analyzed the effect of PFJ overstuffing following TKA without patella resurfacing on the surgical efficacy, without analyzing its clear influencing factors. Based on this, this study collected the clinical data of 168 patients who underwent TKA without patella resurfacing at our hospital during the same period for retrospective analysis.

## Materials and methods

### Clinical data

The study population comprised 168 patients diagnosed with knee osteoarthritis who underwent TKA without patella resurfacing at our hospital from Match 2019 to September 2021. Among the participants, there were 57 males (33.93%) and 111 females (66.07%). Of these, 125 cases had unilateral disease (74.40%), while 43 cases had bilateral disease (25.60%). The average age of the patients was (65.72 ± 4.28) years old, with an average BMI of (28.16 ± 1.20) kg/m^2^. It is important to note that only the first replacement side was considered for patients who underwent bilateral TKA. This study adhered to the Declaration of Helsinki (2013) [10].

### Inclusion and exclusion criteria

Inclusion criteria. (1)Patients underwent primary TKA without patella resurfacing. (2)Patients with complete clinical data, and had clear and standard lateral X-ray films of knee joint in full extension position, both before and after surgery. (3)The prostheses utilized were Genesis II PS prostheses provided by Smith & Nephew manufacturer, and were placed correctly without any anterior or posterior tilt.

Exclusion criteria. (1) Patients with a previous history of fractures of the affected knee joint, PFJ dislocation, and extremely high or low patella, which led to the PFJ to lose normal alignment on X-ray film. (2) Patients who had abnormal enlargement of the patellofemoral joint space caused by massive effusion in the joint cavity or synovial hyperplasia.

### Methods

#### Surgical methods

All TKA without patella resurfacing were performed by the same group of surgeons at our hospital, following a standardized approach outlined as follows. The conventional anterior median incision of knee was performed to separate the subcutaneous tissues, providing access to the knee joint through the medial parapatellar approach. Resection of the distal femur was performed using a combination of the measured resection technique, gap balancing technique [11] and reference technique, ensuring complete resection of the femoral anterior condyle while minimizing excessive bone removal. In cases where varus or valgus deformities were observed in the knee joint, soft tissue balancing was prioritized to achieve proper prosthesis alignment. Surgical intervention involving patellar replacement was not performed; rather, osteophytes and inflammatory tissues surrounding the patella were excised. After denervation using an electric knife, which aims to remove nerve innervation without affecting the patellar articular surface, a bone saw was utilized to appropriately shape the patella and improve its compatibility with the prosthesis. After the operation, the patient was subjected to compression bandaging and standard preventive measures against infection.

#### Measurement methods

**Fig. 1:**
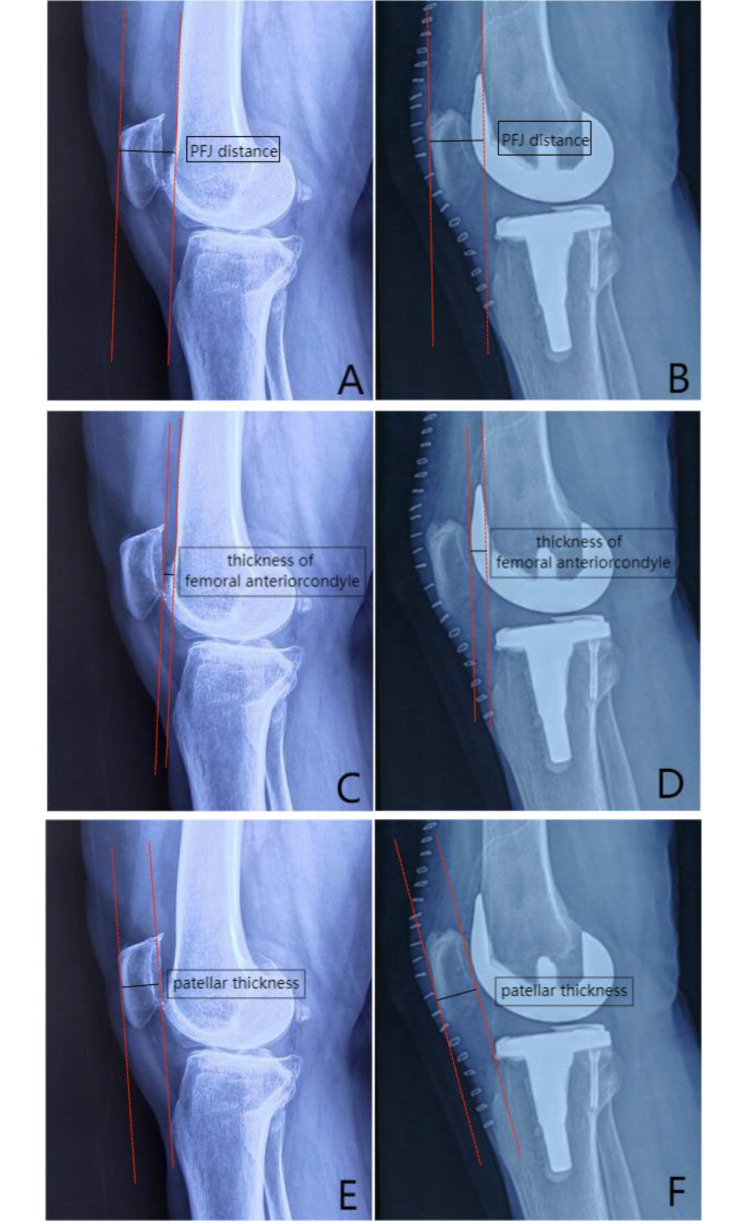
Mapping schematic diagram of each imaging index. Notes: Figure **A**, Preoperative PFJ distance; Figure **B**, Postoperative PFJ distance; Figure **C**, Preoperative thickness of femoral anterior condyle; Figure **D**, Postoperative thickness of femoral anterior condyle; Figure **E**, Preoperative patellar thickness; Figure **F**, Postoperative patellar thickness

The digital X-ray imaging system was adopted to capture knee joint lateral radiographs in full extension positions for patients, within one week before and one week after surgery. The measurements for each index were conducted by the same senior physician who used the PACS software provided by the imaging system, ensuring consistency in methodology [12] (see Table 1; Fig. 1). To obtain accurate values, each index was measured 5 times, and the average value was recorded. Theoretically, as long as the postoperative PFJ distance was greater than the preoperative measurement, it was overstuffing. However, the data of < 1 mm were prone to measurement error. In this study, PFJ overstuffing was defined as a postoperative PFJ distance exceeding 1 mm compared to the preoperative measurement. Conversely, if the postoperative PFJ distance did not exceed this threshold, it was classified as non-overstuffing [13].

**Table 1:** Measurement of each imaging index

Imaging indexes	Measurement range
Preoperative/postoperative PFJ distance	Distance from the anterior cortical line of distal femur to the highest point of anterior cortex of patella
Preoperative thickness of femoral anterior condyle	Distance from the anterior cortical line of distal femur to the highest point of femoral anterior condyle
Postoperative thickness of femoral anterior condyle	Distance from the anterior cortical line of distal femur to the highest point of the anterior condyle of femoral prosthesis
Preoperative/postoperative patellar thickness	Distance from the highest point of anterior cortex of patella to posterior cortical line of patella

#### Technical route

**Fig. 2:**
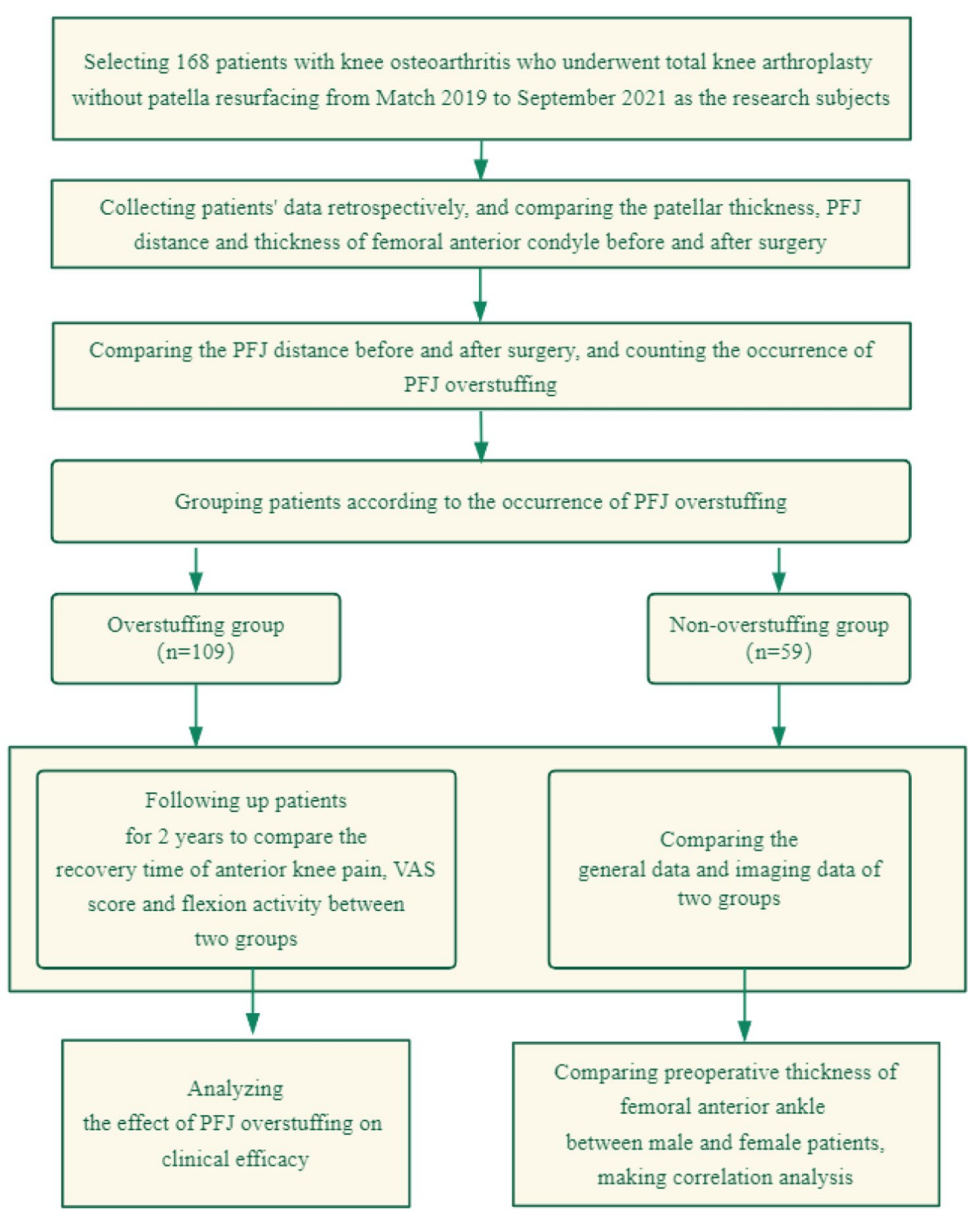
Technical route

This study collected the general data and imaging examination data of patients for retrospective study, including age, body mass index (BMI), gender, sites of disease, occupation and follow-up data in two years (recovery time of postoperative pain, flexion activity of knee joint and VAS score). The technical route of this study is shown in Fig. 2.

### Statistical methods

The statistical software of SPSS26.0 (International Business Machines Corporation, Armonk, State of New York, USA) was used to process all data collected in this study, while GraphPad Prism 7 (GraphPad Software, San Diego, California, USA) was adopted for generating visual representations. The enumeration data were detected by χ^2^ test and presented as [n(%)]. The measurement data were detected by normality test firstly. The measurement data following a normal distribution were analyzed using the t test, whereas data not conforming to a normal distribution were assessed using Mann-Whitney U test, as indicated by Mean ± SD. Spearman rank correlation analysis was used for correlation analysis. A significance level of *P* < 0.05 was used to determine statistical significance.

## Results

### Comparison of imaging indexes before and after surgery

**Fig. 3:**
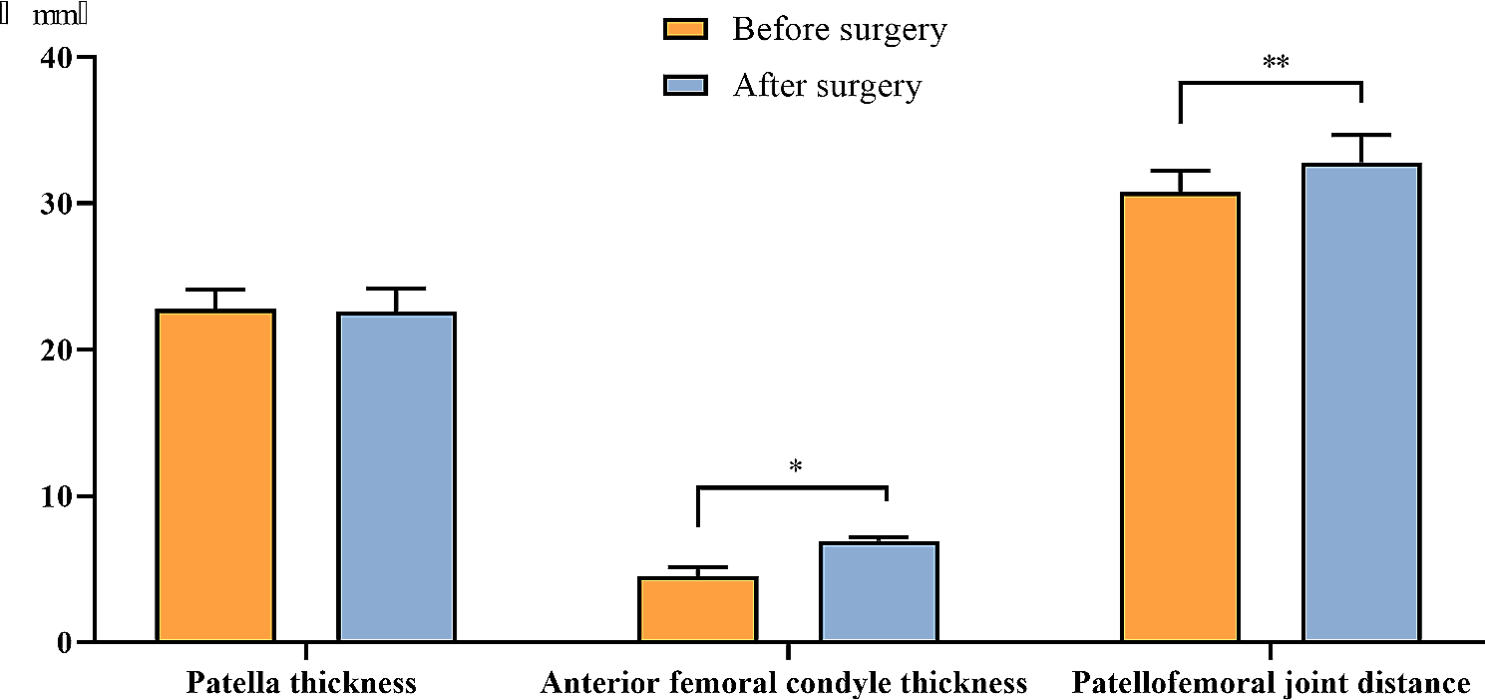
Comparison of imaging indexes of patients before and after surgery [Mean ± SD]. Notes: The patellar thickness, thickness of femoral anterior condyle and PFJ distance in patients before surgery were (22.77 ± 1.36) mm, (4.54 ± 0.62) mm and (30.77 ± 1.48) mm, respectively. The patellar thickness, thickness of femoral anterior condyle and PFJ distance in patients after surgery were (22.59 ± 1.61) mm, (6.92 ± 0.28) mm and (32.80 ± 1.88) mm, respectively. * indicated that the difference in the thickness of femoral anterior condyle of patients before and after surgery was statistically significant (Z=-15.851, *P* < 0.001). ** indicated that the difference in PFJ distance of patients before and after surgery was statistically significant (Z=-9.337, *P* < 0.001)

There was no significant difference in patellar thickness before and after surgery (Z=-1.179, *P* = 0.238). However, the thickness of the femoral anterior condyle and the PFJ distance after surgery demonstrated a significant increase compared to the preoperative measurements (*P* < 0.05), as shown in Fig. 3.

### Occurrence of PFJ overstuffing

Among the 168 patients, 109 cases (64.88%) had PFJ overstuffing. Based on the occurrence of PFJ overstuffing, 168 patients were divided into the overstuffing group (*n* = 109) and the non-overstuffing group (*n* = 59).

### Comparison of patients’ clinical data in both groups

In comparison, the risk of overstuffing in females was significantly higher compared to males (*P* < 0.05). However, no significant differences were observed in clinical data such as age and BMI among the patients (*P* > 0.05). See Table 2.

**Table 2:** Comparison of patients’ clinical data in both groups

Projects	Overstuffing group (*n* = 109)	Non-overstuffing group (*n* = 59)	χ^2^/t/Z	*P*
Age (years old)	65.40 ± 4.34	66.31 ± 4.13	-1.301	0.193
BMI (kg/m^2^)	28.14 ± 1.19	28.18 ± 1.22	-0.576	0.567
Gender			9.402	0.002
Males	28 (25.69)	29 (49.15)		
Females	81 (74.31)	30 (50.85)		
Disease sites			1.153	0.283
Unilateral	84 (77.06)	41 (69.49)		
Bilateral	25 (22.94)	18 (30.51)		
Occupation			0.481	0.923
Worker	51 (46.79)	28 (47.46)		
Farmer	36 (33.03)	21 (35.59)		
Intellectual	15 (13.76)	6 (10.17)		
Others	7 (6.42)	4 (6.76)		

### Comparison of imaging indexes in both groups

The overstuffing group had significantly smaller preoperative thickness of the femoral anterior condyle compared to the non-overstuffing group (*P* < 0.001). However, no significant differences were observed between the two groups in terms of patellar thickness before and after surgery, as well as the thickness of femoral anterior condyle after surgery (*P* > 0.05), see Table 3.

**Table 3:** Comparison of imaging indexes in both groups [Mean ± SD]

Groups	*n*	Patellar thickness (mm)		Thickness of femoral anterior condyle (mm)	
		Before surgery	After surgery	Before surgery	After surgery
Overstuffing group	109	22.73 ± 1.35	22.59 ± 1.63	4.29 ± 0.50	6.96 ± 0.28
Non-overstuffing group	59	22.84 ± 1.39	22.58 ± 1.59	4.99 ± 0.56	6.86 ± 0.27
*Z*		-0.492	-0.080	-6.975	-1.919
*P*		0.623	0.936	< 0.001	0.055

### Comparison of preoperative thickness of femoral anterior condyle between male patients and female patients

**Fig. 4:**
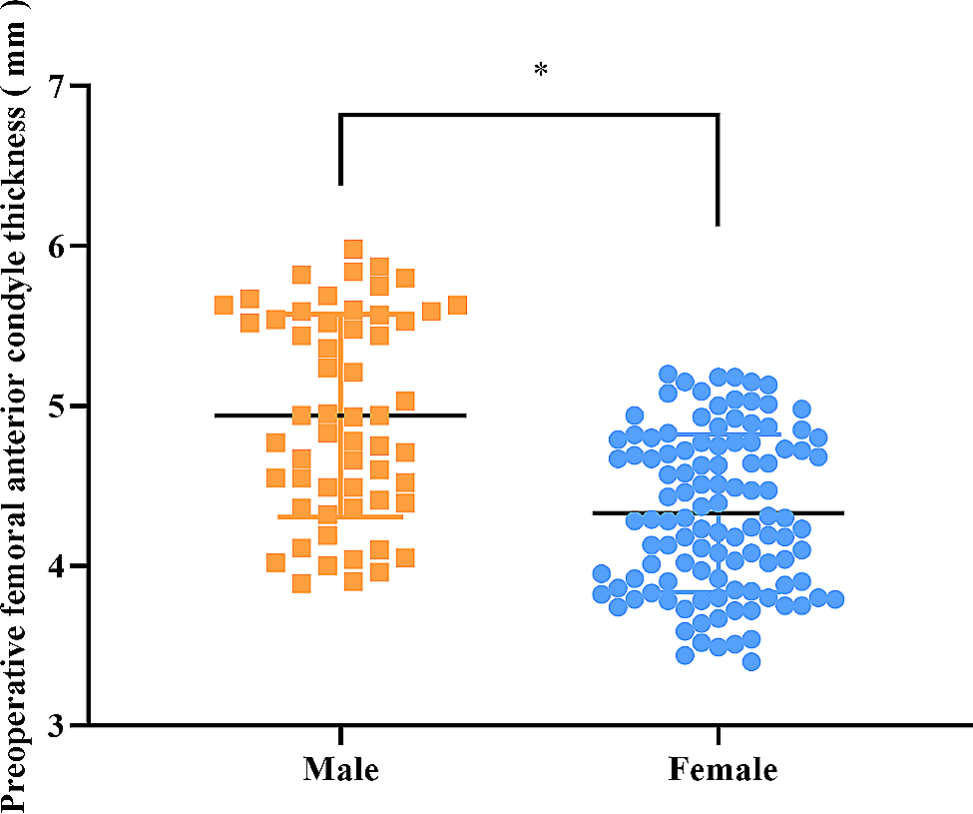
Comparison of preoperative thickness of femoral anterior condyle between male patients and female patients [Mean ± SD]. Notes: The preoperative thickness of femoral anterior condyle in male patients and female patients were (4.94 ± 0.63) mm and (4.33 ± 0.49) mm, respectively. * P indicated that there was a statistically significant difference in the thickness of femoral anterior condyle between female patients and male patients (Z=-5.483, *P* < 0.001)

Figure 4 showed that female patients had overtly smaller preoperative thickness of femoral anterior condyle than male patients (*P* < 0.001).

### Correlation analysis of gender with preoperative thickness of femoral anterior condyle and postoperative PFJ overstuffing

The results of the Spearman rank correlation analysis indicated significant associations within the data. Females tend to have a lower preoperative thickness of the femoral anterior condyle (*r*=-0.424, *P* < 0.001), as well as a positive postoperative PFJ overstuffing (*r* = 0.237, *P* < 0.05). Furthermore, a negative correlation was observed between the preoperative thickness of the femoral anterior condyle and postoperative PFJ overstuffing (*r*=-0.540, *P* < 0.001). See Table 4.

**Table 4:** Correlation analysis of gender with preoperative thickness of femoral anterior condyle and postoperative PFJ overstuffing

Factors	Preoperative thickness of femoral anterior condyle		Postoperative PFJ overstuffing	
	*r*	*P*	*r*	*P*
Gender	-0.424	< 0.001	0.237	0.002
Preoperative thickness of femoral anterior condyle	-	-	-0.540	< 0.001

### Comparison of postoperative symptoms and function between the two groups

Compared with the non-overstuffing group, the overstuffing group had longer recovery time of postoperative pain (*P* < 0.05), and had lower flexion activity at 2 year after surgery (*P* < 0.001). However, there was no significant difference in the VAS score between the two groups at 2 years after surgery (*P* > 0.05), as shown in Table 5.

**Table 5:** Comparison of postoperative symptoms and function between the two groups

Projects	Overstuffing group	Non-overstuffing group	Z/t	*P*
Recovery time of postoperative pain (month)	6.76 ± 2.09	5.71 ± 2.45	2.481	0.013
VAS score (points)	1.37 ± 1.10	1.32 ± 0.94	-0.081	0.935
Passive flexion activity (°)	107.42 ± 13.72	116.36 ± 10.33	-4.206	< 0.001
Active flexion activity (°)	106.34 ± 15.82	115.73 ± 7.36	-3.800	< 0.001
Active flexion activity under load (°)	107.98 ± 12.67	116.63 ± 10.11	-4.022	< 0.001

## Discussion

The PFJ is a complex joint between the patella and femoral trochlear groove [14]. Preserving the patellofemoral anatomical structure leads to physiological patellofemoral kinematics, which can prevent patellofemoral complications and improve the clinical outcomes after TKA [15]. PFJ overstuffing not only causes anterior knee pain, but also affects patients’ efficacy and satisfaction [2, 16–18]. The results of this study show that PFJ overstuffing leaded to an increase in the recovery time of postoperative pain, which may be related to the occurrence of anterior knee pain. Moreover, PFJ overstuffing also caused a decrease in flexion activity. Therefore, this study aims to gather clinical data from 168 patients who underwent TKA without patella resurfacing. The objective is to investigate the factors contributing to postoperative PFJ overstuffing and provide insights for improving surgical plans. Comparative analysis of the clinical and imaging data between the two groups revealed a significantly higher risk of overstuffing in females as compared to males (*P* < 0.05). This suggests that anatomical features related to gender may be associated with postoperative PFJ overstuffing. Based on the findings of the comparison between male and female patients in terms of preoperative femoral anterior condyle thickness, it can be inferred that female patients have smaller thickness of femoral anterior condyle and higher probability of overstuffing after surgery. The above inference is consistent with the previous reports [19, 20]. Despite some manufacturers producing total knee replacement prostheses specifically designed for females, the individualized design of femoral anterior condyle prostheses for females is still not optimal.

The primary reason behind the adverse effects of overstuffing in patients undergoing TKA is the lack of individualized prosthesis design, and the patellar trajectory cannot restore to the physiological value during the surgery [21]. Therefore, analyzing the influencing factors of overstuffing is helpful to reduce the adverse effects of surgery on patients. Some researchers have posited that the amount of osteotomy should be carefully determined in the TKA without patella resurfacing, otherwise the PFJ distance will increase, resulting in overstuffing in patients [22]. Building upon this concept, the present study investigates the impact of patellar thickness and femoral anterior condyle thickness before and after surgery on the occurrence of postoperative PFJ overstuffing. The findings indicate that a negative correlation was observed between the preoperative thickness of the femoral anterior condyle and postoperative PFJ overstuffing (*P* < 0.001). Consequently, it can be hypothesized that a smaller femoral anterior condyle thickness before surgery may represent a risk factor for postoperative PFJ overstuffing.

TKA is still a successful surgery in the case of preserving the patella [23]. However, one clinical study has shown that the shape of prosthesis is different from the conformity of natural patellofemoral joint, which may affect the motor function of PFJ [24]. Under the patella preservation, it is best to use a more anatomical femoral prosthesis design. The Spearman rank correlation analysis conducted in this study reveals a noteworthy association between smaller preoperative femoral anterior condyle thickness and the occurrence of PFJ overstuffing. These findings underscore the importance of carefully considering these factors during patient selection and preoperative planning. It is hoped that this research will raise awareness among scholars regarding postoperative PFJ overstuffing and promote the development of more scientifically sound and safe treatments for patients with end-stage knee arthritis. However, it is important to note that this study employed a retrospective analysis methodology, and the measurement of PACS software and photographic angle position may introduce potential error factors into the collected imaging data. Future research should aim to refine the research design, expand the sample size, and enhance data reliability and overall results.

## Conclusion

In conclusion, there is a substantial likelihood of experiencing PFJ overstuffing following TKA without patella resurfacing, with female patients exhibiting a higher risk compared to males. The small thickness of the femoral anterior condyle may serve as an underlying anatomical factor contributing to postoperative PFJ overstuffing in patients. PFJ overstuffing prolongs the recovery time of postoperative pain, leads to a decrease in flexion activity, and has a certain impact on the efficacy of surgery. However, the primary cause of this issue lies in the lack of individualized prosthesis implantation. Given the limitations of current technology and materials, it is difficult to completely avoid overstuffing resulting from patient-specific differences within a short timeframe. Therefore, during TKA without patella resurfacing, the surgeon should use the prosthesis with thinner anterior condyles and minimize increased tissue tension caused by the surgical procedure to mitigate excessive joint contact pressure, especially for patients with small thickness of the femoral anterior.

## Data Availability

No datasets were generated or analysed during the current study.
